# Ethnobotanical Study of Medicinal Plants Used against Human Diseases in Zuway Dugda District, Ethiopia

**DOI:** 10.1155/2023/5545294

**Published:** 2023-10-18

**Authors:** Moa Megersa, Tesfaye Nedi, Shiferaw Belachew

**Affiliations:** Department of Biology, School of Natural and Computational Sciences, Madda Walabu University, P.O. Box 247, Robe, Ethiopia

## Abstract

**Introduction:**

In Ethiopia, traditional medicine has significantly contributed to the treatment of public health conditions. However, when contrasted with the ethnic diversity of Ethiopians, the number of medicinal plants that have been documented remains limited. As a result, the study aimed to record the medicinal plants used in the Zuway Dugda district communities. *Methodology*. An ethnobotanical study of medicinal plants used by local people of Zuway Dugda district was carried out from February 2020 to November 2020. Semistructured interviews, a market survey, field observations, and group discussions were used to gather pertinent data. One hundred informants (83 males and 17 females) provided data. 76 informants were chosen at random, while 24 key informants were chosen on the basis of recommendations from local elders. Data were analyzed using descriptive statistics, preference ranking, paired comparison, and direct matrix ranking.

**Results:**

A total of 73 plant species, representing 62 genera and 40 families, were collected and identified. Asteraceae, Fabaceae, and Solanaceae had the most species, with each family having 6 (8.22%) species, followed by Euphorbiaceae, which had 4 (5.48%) species. The wild harvest of approximately 49 medicinal plants (or 67.12%) was used. 31 plant species, or 42.47% herbs, were found to be the most commonly used life forms. The most frequently used plant parts were reported to be the leaves, which accounted for 55 species (41.35%) and then the roots 25(18.80%). Headache, toothache, depression, febrile illness, and fever had the lowest ICF values (0.33), while snake bite-related issues had the highest ICF values (0.93). The results of the preference ranking indicated that locals prefer to treat wounds with *Asparagus africanus* Lam. The community used *Cordia africana* Lam. the most, according to direct matrix ranking.

**Conclusions:**

It is essential to combine indigenous knowledge with scientific methods in order to extract the most benefit from medicinal plants. The results of the ICF and preference ranking could be used as a prelude to this. Since *A. africanus* was found to be the most effective wound-healing medicinal plant in the current study, further phytochemical and pharmacological research is recommended.

## 1. Introduction

Since the beginning of time, people all over the world have used plants to treat diseases. Plant-based drugs have been developed as a result of some of the findings from worldwide research to confirm their effectiveness [1]. The use of traditional remedies greatly aided the development of modern drugs. Treatment methods that have been in use for hundreds of years prior to the development and spread of modern medicine make up traditional medicine [2]. The social and cultural traditions of various nations influence the wide range of these practices.

Exercises, spiritual therapies, manual techniques, and medicines derived from plants, animals, or minerals are all included in traditional medicine [3]. These practices, approaches, knowledge, and beliefs are used to treat, identify, or prevent illness in addition to maintain health. Traditional healers and plant-based medicines are heavily used in developing countries to treat human diseases. As a result, ethnobotanical studies are useful for capturing, interpreting, and disseminating information about the ways in which human civilizations and plant diversity interact [4, 5].

Ethnobotanical studies have been conducted by various scholars worldwide. According to recent evaluations, the number of published ethnobotanical studies is increasing annually, both in Ethiopia [6] and elsewhere in the world [7]. Albuquerque et al. [7] claim that additional ethnobotanical research might demonstrate the remarkable advancement of the field as a science. An impressive number of medicinal plants that are used to treat illnesses in humans and livestock have been gathered as a result of a number of studies that were carried out in various regions of Ethiopia. Megersa and Woldetsadik, for example, found 51 plant species that local people in the Damot Woyde district in southern Ethiopia used to prepare traditional medicines [8]. In the Suro Barguda district, 98 different medicinal plants were used to treat human ailments [1]. Usman et al. [9] and Abdela et al. [10] conducted a similar study and recorded 62 and 112 medicinal plants that were used to treat human diseases, respectively. The indigenous people of Ethiopia have reportedly used a number of medicinal plants, which suggests that they have extensive knowledge of these plants and their applications [8].

According to these studies, the country's knowledge of medicinal plants is vanishing for a variety of reasons. Indigenous knowledge of medicinal plants is passed down from one generation to the next orally. In this way, the knowledge transfer system may lose fundamental information about how to use plants and their parts, drug preparation techniques, and other things [11]. The primary threats to medicinal plants in Ethiopia were also identified as the expansion of modern education, religion, agricultural expansion, urbanization, overexploitation, and firewood collection. Local people of Ethiopia had the knowledge of conserving medicinal plants though many authors reported the effort is minimal. The Guji Oromo has their belief (*Waaqeffannaa*) which has a positive contribution to medicinal plants management. In this indigenous belief, cutting big trees is out of forbidden, because when it falls, it has a very huge sound and power which would disturb God (*Waaqa*) and all humans closer to the area as well [1]. The study conducted in Wayu Tuka district showed that local healers do not conserve medicinal plants very well, and they preferred to collect them from wild stands when patients visit them. Local people conserve medicinal plants of the public domain in home gardens where *Ocimum urticifolium* hort. ex Benth and *Ruta chalepensis* L. are among the frequently grown medicinal plants [11]. The local people of Asagirt district use some methods to conserve and protect medicinal plant species. The highest conservation method recommended by the local people was home gardens, followed by plantation in mosques and churches [12].

Taking into account the diverse cultures, ethnicities, types of vegetation, and climate zones, the country has very little documented ethnobotanical information about medicinal plants. The Zuway Dugda district in Oromia is one of the areas that receive less attention. Hence, the aim of the present study was to document the medicinal plants and associated knowledge of the communities in Zuway Dugda district and the threats currently affecting medicinal plants. The specific goals of the study were to evaluate how the district's local communities conserve medicinal plants for use in the treatment of human diseases.

## 2. Materials and Methods

### 2.1. Description of the Study Area

**Figure 1:**
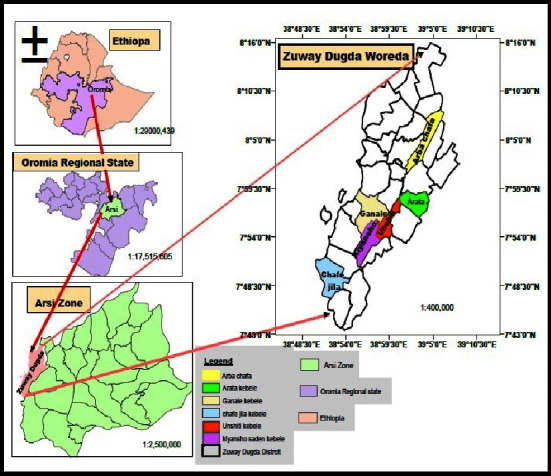
Map of Zuway Dugda district, Arsi zone, Oromia, Ethiopia showing surveyed kebeles.

Zuway Dugda district is situated at 7°44^″^N and 8°16^″^ N and 38°50^″^ E and 39°8^″^ E. It is located at about 156 km south east of Addis Ababa, in East Arsi Zone of Oromia Regional state Ethiopia. The district's capital, Ogolcho, is approximately 46 kilometers away in Zonal Capital Asella (Figure 1).

### 2.2. Topography

The altitude of the district ranges from 1600 to 1800 meters above sea level and the district has various topographic features, 65% (53,388,615 ha) of the land areas is plain, 15% (12,320,449 ha) hilly areas, and cliffs and valleys account 12% (9,856,359 ha) and 8% (6,570,906 ha), respectively [13].

### 2.3. Climate

**Figure 2:**
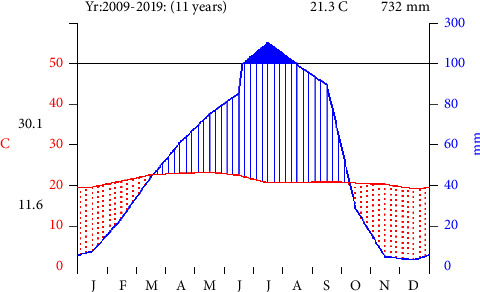
Climadiagram of the study area from 2009 to 2019 at Batu station. Data source: National Meteorological Service Agency.

According to Zuway Dugda district administration office [13], the climate of the area is classified traditionally into two main agroclimate zones; “Baddadaree (Woina dega) and Gammojjii” (Kola). The total land mass of the district, about 89.65% (73,635,222 ha) fall under “Gammojjii” while 10.35% (8,501,111 ha) is categorized under, “Baddadaree” (Woina dega). The rainy season (winter) is traditionally called, “Ganna” and dry season (summer) is, Bona “Ganna” (rainy season) is extends from June to August with the highest peak in July and August. The highest monthly rainfall in July and the lowest May. The maximum mean temperature was recorded in February and May (32°C). The minimum mean temperature was recorded in October and November (19°C). In general, the mean annual temperature and mean annual rainfall of the Zuway Dugda district is (21.3°C) and (732 mm), respectively (Figure 2).

### 2.4. Population

The population of the Zuway Dugda district in 2023 projected to be 175,208 according to CSA [14], with 49.7% (87,145) of those people being male and 50.2% (88,063) being female. The Oromo people make up the majority of the community in the district. Afan Oromo is a widely spoken language in the district.

### 2.5. Educational and Health Services

There are three nongovernmental kindergarten schools in the district [15]. The district also has six first-cycle elementary schools (ages 1–4), 39 second-cycle elementary schools (ages 5–8), five secondary schools (grades 9–12), and one private high school. However, the district only has one government school for technical and vocational education.

There are thirteen health posts, six governmental health centers, and four nongovernmental clinics [16]. In general, the district's health issues are connected, either directly or indirectly, to issues with sanitation, poor diet, a lack of potable water, and poor house condition. In this region, high fever is a common symptom of acute febrile illness, whose cause is unknown. The most prevalent human diseases in the district are acute febrile illness, upper respiratory tract infection, pneumonia, wound and injury (accident), gastrointestinal tract infection, diarrhea, sexually transmitted diseases, urinary tract infection, skin infection, and joint disease.

### 2.6. Study Sites and Informant Selection Techniques

Study sites and informants were selected based on the information gathered from Zuway Dugda district administration, health and agricultural offices. Accordingly, six (6) kebeles (the smallest administrative unit), namely, Kiyansho, Unisheti, Arba chefe, Genale, Chefe jile, and Areta cufa were selected from a total of 30 kebeles for data collection with purposive sampling method based on their agroecological conditions, recommendations of local elders, the availability of traditional medicine practitioners, and vegetation cover. Kiyansho, Genale, Chefe jile, and Unisheti were selected from low land agro climatic zones and Arba chefe and Areta cufa from midland. From the six selected kebeles, a total of 100 informants (83 males and 17 females) were selected randomly. Based on the information they provided during an interview, 24 key informants (22 males and 2 females) were selected. Each of the sampled Kebele health officers, local authorities, and knowledgeable elders provided information about healers who were also considered as a key informant.

### 2.7. Ethnobotanical Data Collection

**Figure 3:**
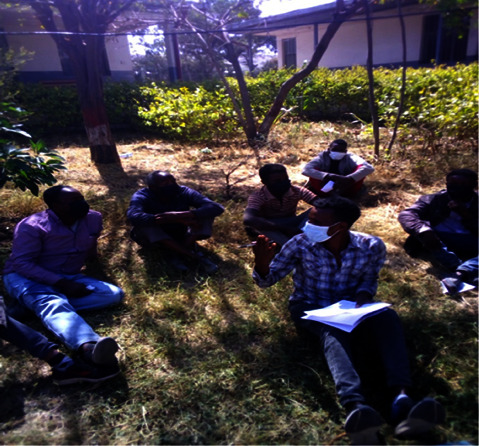
Group discussion held on threats and conservation to medicinal plants with informants' photo taken by Fikadu, (2020).

Ethnobotanical data were collected from February 2, 2020 to November 2, 2020 through semistructured interviews, guided field walks, observations, and focus group discussions (Figure 3). The semistructured interviews were conducted using English-language questions that had been translated into Afan Oromo. The items of the interview are based on the informants' gender, age, occupation, educational level, religion, and category (healer or general informant). The uses of medicinal plants, which include local names, diseases treated, parts used, preparation methods, administration routes, dosage, habitats, and how local people conserve medicinal plants, are also included in the list of questions.

Guided field walks were conducted with interviewees and other local indigenous people to look for additional wild medicinal plants and gather specimens for vouchers. All interviews were conducted in the Afan Oromo language, which is spoken by the people who live in the area under study.

#### 2.7.1. Market Surveys

**Figure 4:**
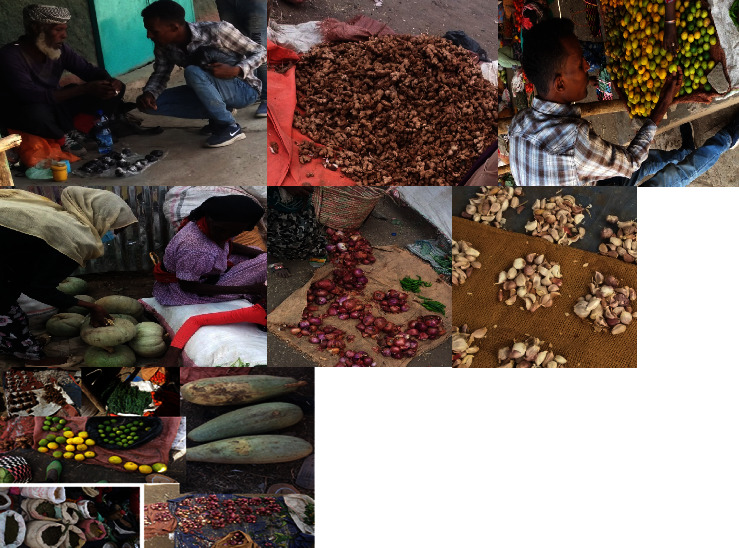
The two market surveys in the district (Chefe Jila and Ogolcho market) (photo taken by Takele Gebregiorgis (2020)).

In Chefe Jila and Ogolcho towns, two markets, Jila and Abura, were chosen for the current study (Figure 4). In order to record the names of the medicinal plants, prices, and other aspects of herbal medicines sold in the local markets of the study area, market surveys were conducted. In accordance with the recommendations outlined by Martin [4] and Alexiades [17], observations in the two markets and interviews regarding aspects of wild plant products were carried out in order to identify the medicinal plants that are sold in the market.

#### 2.7.2. Voucher Specimen Collection and Identification

During a guided field walk, the voucher specimens were collected, and the plants were numbered, pressed, and dried for identification. Specimens were identified both in the field and in the Madda Walabu University mini herbarium. In addition, various Ethiopian and Eritrean floras were used in the identification process [18–24].

### 2.8. Data Analysis

#### 2.8.1. Descriptive Statistics

The information on medicinal plants, use, and related knowledge was analyzed and summarized using a descriptive statistical approach, such as percentage and frequency. Descriptive statistics were used to examine the most important information on medicinal plants that locals reported, including the most frequently used plants, medicinal value, application, methods of preparation, route of application, diseases treated, and parts utilized and habits. For analysis, a Microsoft Excel spread sheet was employed. MS Excel spread sheet was used for analysis.

#### 2.8.2. Informant Consensus Factor

The diseases of the study area were grouped into various categories based on the site of occurrence of the disease, condition of the disease, and treatment resemblance of the disease to the local people. In order to evaluate the reliability of information during the interview, informants are contact at least two times for the same ideas and the validity of the information is proving and record. Only the relevant ones are analyzed. The informant consensus factor (ICF) was calculated for each category to identify the agreements of the informants on the reported cures for the group of ailments. The ICF was calculated as follows: number of use citations in each category (nur) minus the number of species used (nt), divided by the number of use citations in each category minus one [25]. The factor provides a range of 0–1, where a high value acts as a good indicator for a high rate of informant consensus. Here, ICF is informant consensus factor.

(1) $$\begin{array}{c} ICF=ur\text{ is number of use citation},\\ n_{t}\text{ is number of species used}. \end{array}$$

#### 2.8.3. Preference Ranking

Following Martin [4], a preference ranking was computed for the eight most important medicinal plants used to heal wounds. Ten of the key informants were shown these plants with the goal of getting their own preferences for the best recommended therapeutic herbs. The medical plant thought to be most capable of curing the ailment has the greatest value (five), while the one thought to be least capable has the lowest value (1). Each species' value was added together, and each species' ranking was established using the overall score.

#### 2.8.4. Paired Comparison

A pairwise comparison method was used to determine the relative importance of plant species, where items were presented in pairs and decisions were made by individual respondents about the relative importance of one of the items in the pair [4]. Based on Martin's recommendations [4], a pairwise comparison of the popularity of five medicinal plant species used to treat febrile illnesses was calculated. Thus, seven key informants were randomly selected by coin toss to independently demonstrate answers to five pairs of traditional medicinal plants known to treat febrile illnesses. A list of pairs of selected items containing all possible combinations is created, the order of the pairs and the order within each pair are randomized, and then, each pair is presented to the selected informant and the responses were recorded. Total scores are summarized and ranked. The total number of possible pairs (10) was obtained by applying the following formula:

(2) $$\begin{array}{c} \frac{N\;\left(n‐1\right)}{2},\text{where }n\text{ is the number of items}. \end{array}$$

#### 2.8.5. Direct Matrix Ranking

A direct matrix ranking was performed to compare the multipurpose medicinal plants commonly reported by informants after cotton [26]. Based on the relative benefits obtained from each plant, six multipurpose tree species across medicinal plants and seven uses for these plants are listed. To assign a use score to each attribute (5 = best, 4 = very good, 3 = good, 2 = less used, 1 = least used, and 0 = less used), ten key informants were selected. Based on information gathered from informants, average values for each use of the species were obtained and the values for each species were summed and ranked.

## 3. Results

### 3.1. Medicinal Plants of the Study Area

A total of 73 medicinal plants were reported to be used to treat human diseases by local informants in the study area. These plants are distributed in 62 genera and 40 families. The most diverse families in terms of species composition were Asteraceae, Fabaceae, and Solanaceae, each with 6 species (8.22%), Euphorbiaceae with 4 species (5.48%), and Cucurbitaceae, Boraginaceae, and Lamiaceae with 3 species (4.11%). Amaryllidaceae, Apiaceae, Brassicaceae, Meliaceae, Ranunculaceae, Rhamnaceae, and Rutaceae represented by 2 species each (2.74%). The remaining families are each represented by one (1.37%) species (Table 1). Of the medicinal plants used for human diseases, 49 (67.12%) species were collected from wild vegetation and 24 (32.88%) species were collected from home gardens.

### 3.2. Growth Form and Plant Parts Used to Treat Human Diseases

**Figure 5:**
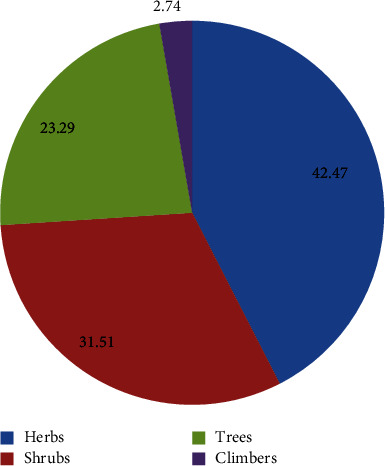
Growth forms of medicinal plants in the study area.

A growth habit analysis of medicinal plants showed that 31 species (42.47%) of herbs represented the highest percentage, while shrubs represented by 23 species (31.51%) (Figure 5).

**Figure 6:**
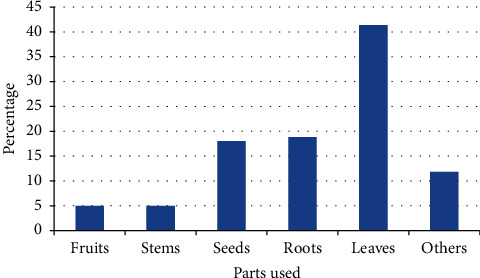
Proportion of plant parts used for preparation of medicines.

Regarding plant parts used, 55 (41.35%) were leaves and 25 were roots, accounting for 18.8%, which were mainly used to treat human diseases (Figure 6).

### 3.3. Conditions of Preparation, Dosage, and Route of Administration

Local people in the study area prepare herbal remedies for various ailments, and medicinal plants are available fresh, dried, or fresh and dried. It was reported that approximately 59.4% were used in fresh form, 35.34% were made from dried plant parts, and 5.26% were made from both dried and fresh plant parts. Among the collected medicinal plants, preparations were mainly used in the study area, mainly single and different types of plant parts. Of the two different formulations used by local populations in the study area, 84.96% were associated with a single plant species and 15.04% were associated with different plant species.

Local people of the study area used different units of measure and dosing durations to determine dosage. Local units such as finger length for (bark, root, stem, etc.), various measuring materials (spoon, coffee cup, teacup, glass cup, etc.), and numbers (leaves, seeds, fruits, tubers, rhizomes, flowers, latex, etc.) were used to estimate and correct the dose of the drug. However, these measurements are not precise enough to determine exact doses. Remedies were administered without exact dosages, but locals used traditional units of measurement, such as spoons, coffee cups, teacups, counts or numbers, tin can, and glasses to determine dosage particularly taken through mouth, nose, ears, and eyes.

Local people in the study area were administered traditional medicine mainly orally. Oral accounts for 63.91%, followed by dermal (23.31%), nasal (10.53%), and other (2.25%) (Table 2). Locals also reported that various additives were administered when administering traditional medicines. The remedies were taken with coffee, milk, honey, water, salt, tea, red teff powder, sugar, oil, and butter.

### 3.4. Transfer of Knowledge on Medicinal Plants

Ethnomedicinal knowledge is concentrated among elders and their relatives in the community and is difficult to transfer the knowledge from older to younger generations. The informants of the current study who were interviewed indicated that they obtained their knowledge of medicinal plants from a variety of sources, mostly from family (71%), relatives and friends (10%), and others got it from a healer (4%) through the transfer of specific knowledge about medicinal plants. Knowledge of the identification and handling of medicines with the ingredients used and ecology is mainly related to oral knowledge transfer by locals and the elderly. Oral transmission of knowledge about such medicinal plants is declining and knowledge about medicinal plants is disappearing. Indigenous knowledge of medicinal plants encountered problems in passing on this knowledge and the practice of traditional medicine, according to informants, as younger generations were unwilling to acquire knowledge of traditional medicinal plants.

### 3.5. Ranking of Most Important Medicinal Plants

#### 3.5.1. Informant Consensus

Of the 73 medicinal plants collected in the study area, the use of some medicinal plants to treat human diseases was frequently reported, while the use of others was less reported. Among the medicinal plants reported in the study area, *Ocimum lamiifolium* Hochst. was named by 57 respondents, followed by *Aloe pubescens* Reynolds, 54 respondents named the plant species (Table 3).

#### 3.5.2. Informant Consensus Factor (ICF)

Diseases in the study area were grouped into different categories based on the location of disease occurrence, disease status, and similarity of disease treatment to local populations. Medicinal plants believed to be effective in treating specific ailments had higher ICF scores, indicating that these ailments were more common than those with lower ICF (Table 4). Snake bites had the highest ICF value (0.93) in the study area due to disease incidences. Depression, febrile illness, fever, headache, and toothache (0.33) had the lowest ICF scores in the study area.

#### 3.5.3. Preference Ranking

Wound was one of the most common diseases in the study area. Eight medicinal plants have been described as effective in treating wounds, and ten leading informants ranked these eight plant species based on their perception of their level of effectiveness. Thus, *A. africanus* was the most preferred medicinal plant for wound healing and *Solanum marginatum* L.f. was the least preferred medicinal plant (Table 5).

#### 3.5.4. Paired Comparison

For medicinal plants identified by informants to treat febrile illnesses, paired comparison between five medicinal plants was performed. Seven key informants participated in this activity. Hence, *O. lamiifolium* ranked first, followed by *B. aegyptiaca*, and the least preferred medicinal plant for treating febrile diseases was *Cynoglossum lanceolatum* B.Heyne ex Wall (Table 6).

#### 3.5.5. Direct Matrix Ranking

In the study area, plant species with multiple uses were studied. Key informants selected 10 plant species with multiple uses. Common uses include medicine, firewood, charcoal, construction, fencing, and edibles. This finding indicates that *C. africana* ranked first for its multiple uses, followed by *Olea europaea* subsp. *Cuspidata* (Walland G.Don) Cif.; on the other hand, *Carica papaya* L. came last and was the least frequently used in the study area (Table 7).

#### 3.5.6. Marketable Medicinal Plants

As explained by the respondents, traditional medicines prepared by healers from plants or parts of plants are sold at home rather than on the open market due to the preference of local people. Medicinal plants marketed on the open market as a spice for nonmedicinal purposes *A. sativum*, *A. cepa*, *Artemisia abyssinica* Sch.Bip., *Capsicum annuum* L., *Coriandrum sativum* L., *Lepidium sativum* L., *Nigella sativa* L., *O. basilicum*, *Trigonella foenum-graecum* L., and *Zingiber officinale* Roscoe, food (*Brassica carinata* A.Braun, *B. nigra* (L.) K.Koch, *Cucurbita pepo* L. and *L. usitatissimum*, *C. papaya* and *Citrus limon* (L.) Osbeck).

#### 3.5.7. Threat and Conservation of Medicinal Plants of Study Area

*(1) Threat of Medicinal Plants*. Agricultural expansion, charcoal production, construction, and firewood collection were common threats to medicinal plants. Informants ranked agricultural expansion (19.13%) as the most common threat to medicinal plants, followed by charcoal production (18.7%) (Table 8).

*(2) Conservation of Medicinal Plants.* Efforts to conserve medicinal plants within the district were classified as low. Some traditional practitioners have begun to grow medicinal plants in home gardens, but their efforts have been minimal. Medicinal plants grown in home gardens include *A. cepa, Daucus carota* L. and *A. cepa*, *V. abyssinica*, *C. annum*, *L. sativum*, *R. chalepensis* L., *Cymbopogon citratus* (DC.) Stapf, and *Rhamnus prinoides* L'Her.

## 4. Discussion

### 4.1. Medicinal Plants of the Study Area

A total of 73 medicinal plants belonging to 62 genera and 40 families were reported to be used for the treatment of human diseases by local informants in the study area. The number of medicinal plants reported and their use by the local population of the area shows the depth of local indigenous knowledge of medicinal plants and their uses. The knowledge and practice of a large number of medicinal plants by the people of the Zuway Duguda district shows that the indigenous people of the study area still rely on traditional medicines derived from plants. Asteraceae, Fabaceae, and Solanaceae are the most commonly reported medicinal plants. Fabaceae has also been described as a major medicinal family in ethnobotanical studies elsewhere in Ethiopia [1, 27, 28]. Similar studies reported Asteraceae as the major family in Ethiopia [8, 12, 29, 30], and Solanaceae was mentioned in the work of Mesfin et al. [31]. In contrast, other studies found Lamiacae [32] and Euphorbiaceae [33] to be dominant over others. Two families (Asteraceae and Fabaceae) are most common in the Ethiopian flower region. In addition, the Asteraceae is dominant because its members are known for their scent properties and are widespread in nature. This dominance may be due to the herbaceous lifestyle of the family. The findings are reasonable since the mentioned families are represented by higher number of species in the Ethiopian flora.

The majority of medicinal plant species are obtained from wild habitats. Of the 73 medicinal plants collected, majority were obtained from the wild. The results indicate that communities tend to rely more on wild-collected medicinal plants than on home gardens. Our results are consistent with the general pattern reported in many ethnobotanical studies conducted in Ethiopia [9, 10, 34, 35] and elsewhere in the world [36, 37]. These findings call for the urgent need of medicinal plants conservation in home gardens or by other approaches.

### 4.2. Growth Form and Plant Parts Used

The most common herbal remedies used by the people of the Zuway Dugda district were derived from herbs. The report indicates that herb use was easy and accessible, as the herb grows primarily on roadsides and in home gardens at the study site. Similar results were reported by previous ethnobotanical studies [10, 37–39]. In contrast, other studies have found shrubs to predominate in traditional medicine preparations [1, 40, 41]. The shrub dominance, as shown by Eshete and Molla [1], may be due to the high prevalence and impact resistance of shrubs in the study area.

In terms of parts of use, leaves were the most commonly used for treatment within the study area compared to other parts. Our results were consistent with previous studies reporting leaves as a major medicinal plant part in various other study areas [29, 34, 37, 42]. Given the most abundant leaves used for medicinal purposes in the study area, the risk of destroying medicinal plants was considered minimal. The reason for the frequent use of leaves may lie in their function as centers of photosynthesis and other metabolic activities, thus most secondary metabolites are formed in leaves [43]. In addition, leaves are considered the simplest and most accessible part of the plant for medicinal preparations, rather than other parts of the plant [37]. Other studies on aspects of ethnobotany have shown that the root is the most commonly harvested part of the plant used to make traditional medicines [33, 36, 44]. The use of root for traditional medicine preparation may have a detrimental impact on plant species and thus a wise approach of collection or conservation of such medicinal plants is vital.

Most traditional medicines are primarily made from single plants or plant parts. This finding is similar to others reported in Ethiopia [39, 45]. Other similar studies have reported the use of mixtures of different species to treat disease, rather than using a single species [46]. This difference may be due to different sources of information. The present study was conducted across communities, whereas the previous study was conducted with traditional healers shown to be the most common [11].

### 4.3. Route of Administration and Dosage of Traditional Medicine

The most common route of administration was oral followed by dermal. Reports similar to our study showed that oral administration of drugs was common, followed by dermal and nasal administration [1, 9, 28]. In other studies, topical application is an important method of administering herbal medicines used to treat various external diseases [37]. For this reason, oral and dermal administration allows rapid physiological responses between prepared drugs and pathogens, enhancing their therapeutic potential [47]. This may be because the two pathways are more effective, as the prepared drug reacts quickly with the pathogen's physiology and enhances its healing power [39]. Frequent occurrences of internal disease in the study area may also be a further indication.

Regarding doses, local people in the study area used a variety of units of measurement, including coffee cup, fingertip, teaspoon, glass, and palm. Traditional units of measurement such as spoons, coffee cups, teacups, counts or numbers, tin cans, and glass cups are commonly used to determine dosage. In a similar study, Eshete and Molla [1] reported that dosages prescribed for various ailments are based on patient age, sex, and physical appearance. This indicates that there is no standardized measurement, except that it is determined based on the long experience of traditional healers [1]. Similar approaches have been reported in different parts of the country [32, 39]. Lack of precise dosage is known to be a major drawback of using traditional medicine [48]. This discrepancy could cause problems for patients due to overdose effects. However, as reported by Megersa and Tamrat, local healers give various additives as antidotes to side effects [32].

### 4.4. Ranking of Most Important Medicinal Plants

#### 4.4.1. Informant Consensus Factor (ICF)

The results of the study showed that common diseases in the study area had higher informant consensus factors. Snakebite had the highest ICF score, followed by fever and headache, due to the high incidence of illness in this region. The highest calculated ICF value indicates the best agreement among informants regarding the use of a medicinal plant species to treat a particular disease [39]. High ICF values are important for naming plants of particular interest when searching for bioactive compounds [49]. In addition, plants achieving higher informant consensus scores were thought to exhibit better efficacy when the treatment included a biologically active ingredient compared to plants with lower informant consensus scores. Different results have been reported by researchers in different parts of the world. Thus, the highest ICF value for bone and joint disease was recorded in Rif, northern Morocco [42], and gastrointestinal disease recorded his highest ICF value reported by Eshete and Molla in Suro Barguda district, Ethiopia [1]. The difference of findings among studies could be due to the difference in the prevalence of diseases in the study areas.

#### 4.4.2. Preference Ranking

When there are different species prescribed for the same health problem, people show preference for one over the other. The analysis of preference ranking exercise on medicinal plants that were reported to be used against wounds showed that *A. africanus* was the most preferred species followed by *D. viscosa*. This finding showed that wound was most frequently reported human disease in the district. Similar studies conducted by Jima and Megersa [33] in Berbere district reported different medicinal plants used for wound treatment. This finding may show as the medicinal plant knowledge varies among different communities and also show the importance of such studies to discover a modern drug for diseases.

#### 4.4.3. Direct Matrix Ranking

Direct matrix ranking results showed that *C. africana* ranked is a versatile plant species. The results of Megersa et al. [11] showed *C. africana* as the highest multipurpose species in the Wayu Tuka district, West Wellega Zone of Oromia, Western Ethiopia, and Goro district Bale Zone of Oromia [9]. Local communities in the Hulet Eju Enese district also use *C. africana* for various purposes [41]. On the other hand, various plant species have been reported to rank among the highest for multi-purpose use in various communities in Ethiopia. For example, *O. europaea* subsp. *cuspidata* has been described as the best plant species used for various purposes in communities of Sedie Muja district [39], *Prunus africana* (Hook. f.) Kalkman [1] in Suro Barguda district. A wide range of plant species are most threatened if appropriate conservation, management, and sustainable usage measures are not taken [34]. For example, *C. africana* is endangered due to its multiple uses, as evidenced by its sparse distribution in various regions of Ethiopia [11, 31]. Therefore, it is attracting attention as a native tree species. Conservation priority is therefore given by using domestic in situ and ex situ conservation [50]. Additional conservation measures are urgently needed to prevent future extinctions of other reported multipurpose plant species [51].

## 5. Conclusions

Our results indicate that plant species play an important role in the treatment of various human diseases in the Zuway Dugda district of Ethiopia. As a result of this study, 73 medicinal plant species were recorded. Asteraceae, Fabaceae, and Solanaceae are the three families with the highest percentage of medicinal plants in the study area. The number of medicinal plant species recorded in the study area is a good indicator of the potential of medicinal plants for the development of modern medicines. To get the most out of medicinal plants, it is essential to implement indigenous knowledge in a scientific way. For this, the ICF and preference ranking results can be used as precursors. In the present study, *A. africanus* was the most preferred medicinal plant for treating wounds. Therefore, it is recommended to conduct phytochemical and pharmacological studies to confirm the wound-healing properties of the plant. Medicinal plants in the study area are threatened by various factors, with agricultural expansion accounting for the largest share. Local communities play an important role in preserving medicinal plants around their home gardens, but it is clear that most plant species are where they have been collected from wild habitats. The effort involved is minimal. Therefore, the focus of conservation should be on the most threatened and preferred medicinal and multipurpose plant species.

## Figures and Tables

**Table 1:** Taxonomic diversity of medicinal plants in Zuway Dugda district.

Family names	No of species	Percentage	No. of genera	Percentage
Asteraceae	6	8.22	4	6.45
Fabaceae	6	8.22	3	4.83
Solanaceae	6	8.22	5	8.06
Euphorbiaceae	4	5.48	3	4.83
Cucurbitaceae	3	4.11	3	4.83
Lamiaceae	3	4.11	3	4.83
Boraginaceae	3	4.11	3	4.83
Amaryllidaceae	2	2.74	1	1.61
Amaranthaceae	2	2.74	1	1.61
Apiaceae	2	2.74	2	3.22
Apocynaceae	2	2.74	2	3.22
Brassicaceae	2	2.74	2	3.22
Meliaceae	2	2.74	1	1.61
Ranunculaceae	2	2.74	2	3.22
Rhamnaceae	2	2.74	2	3.22
Rutaceae	2	2.74	2	3.22
Other 24 families	26	35.62	23	33.33
Total	73	100	62	100

**Table 2:** List of medicinal plants used to treat human in the study area.

Family	Scientific name	L. name	Hb	Ht	CP	PU	Ut	Mode of preparation and dosage	RA	Cno.
Acanthaceae	*Justicia schimperiana* (Hochst. ex Nees) T. Anders	Dhumuug aa	Sh	W	F	L	Rabies	Crushed, squeezed, and mixed with milk given 1/2 coffee cup	O	TNO22
							Rheumatism	Mixed with *B. antidysenterica*, *C. macrostachyus*, *Clausena anisata* (Willd.) Hook.f. ex Benth., *Pycnostachys abyssinica* Fresen boiled and washed for 3 days	D	

Amaryllidaceae	*Allium cepa* L.	Qullubbii diimaa	H	HG	F	S	Hypertension	Crushed seed of *A. cepa* and immersed in water filtrated by clean cloth and drunk before food for 1 day	O	TN065
	*A. sativum* L.	Qullubbii adii	H	HG	D	Bu	Colds	Pounded of blub mixed with honey and eaten 2-3 teaspoon for three days	O	TN059
							Malaria	Bulb is pounded, mixed with the crushed fresh leaves of *R. chalepensis*, *S. nigrum* and applied external part		
					D	Bu	Wound	Pounded of bulb together with seed of *L. sativum* and *Ricinus communis* L. and tied on the wound	D	

Aloaceae	*Aloe pubescens* Reynolds	Argiisa	H	W	F	Lat	Fire burn	The latex is painted on the wound part	D	TN07

Amaranthaceae	*Achyranthes aspera* L.	Darguu	H	W	F	L	Body swelling	Leaf is squeezed and creamed on swollen part	D	TN019
							Wound	Leaves are pounded and tied on the wound		
							Nasal bleeding	Fresh leaf of *A. aspera* is squeezed and its juice is dropped in to the nostrils to stop bleeding	N	
						R	Stomachache	Root is chewed and swallowed during feeling of ache	O	
	*Achyranthes* spp. L.	Darguu arbaa	H	W	F	L	Pneumonia	Fresh leaf are pounded, mixed with water and drunk in the morning	O	TN020

Anacardiaceae	*Schinus molle* L	Qundobar baree	T	W	F	L	Problems in throat	Fresh leaves of *S. molle* are chewed on surfaces of throat and holding	O	TN068

Apiaceae	*Foeniculum vulgare* Mill.	Insilaalii	H	W	F	L	Urine retention	Pounded fresh leaf of *F. vulgare* and *J. schimperiana* are Mixed with water and drunk	O	TN039
							Stomachache	Fresh leaf of *F. vulgare*, garlic and pepper are grounded, mixed with water and given with katicala		
	*Daucus carota* L.	Kaarotii	H	HG	F	R	Night blindness	The fresh root of *D. carota* is eating repeatedly	O	TN041

Apocynaceae	*Carissa spinarum* L.	Agaamsa	Sh	W	D	R	Evil sprit	Root of *W. sominifera* pounded together added to fire and smoke to the patient	N	TN05
							Stabbing pain	Root is pounded, boiled in water and is drunk using a glass	O	
							Gonorrhea	Root together with dried root bark of *Euclea racemosa* L. crushed, boiled, add goats milk and is drunk after cooling		
					F		Malaria	Fresh root is pounded, insert into cold water, wait for a day and is drunk		
					F	R	Wound	The bark is pounded & applied on wound for three consecutive days	D	
					D	R	Evil eye	The smoke of pounded roots is inhaled	N	
						L	Headache	Dried leaf of *C. spinarum i*s pounded and the smoke is used as treatment for headache		
					F	L	Stomachache	Leaf of *C. spinarum* is pounded mixed with honey. Two-three spoon is taken early in the morning before breakfast	O	
	*Acokanthera schimperi (*A.DC.) Schwein	Qaraaruu	Sh	W	F	L	Leprosy	Leaves of this plant are pounded together with *B. antidysentetica*, *Rumex abyssinicus* jacq. then mixed with butter and applied on the skin	O	TN054

Asparagaceae	*Asparagus africanus* Lam.	Sariitii	Sh	W	D	R	Placenta retention	Root is pounded; warm in water then mixed with honey and is drunk	O	TN062
					F	R	Wound	Fresh root is pounded together with leaves of *D. viscosa*, mixed with butter and applied on the wound		
					F	L	Malaria	Leaf is crushed, mixed with milk and taken one coffee cup every morning until the individual goes from the malarious area		

Asteraceae	*Echinops angustilobus* S. Moore	Qoree harree	H	W	D/F	R	Headache	Fine root powder is mixed with pounded *A. sativum* and taken pasted with honey	O	TN058
	*Echinops hispidus* fresen.	Kaabariic hoo	H	W	D	R/St	Evil eye	Crushed and placed on fire and inhaled by all householders	Sm	TN042
	*Artemisia abyssinica* sch. Bip.ex A. Rich	Harrittaa	H	HG	F	wh	Evil eye	Leaf concoction together with root of *E. kebericho* is added to a burning fire and smoked to the patient	Sm	TN033
	*Artemisia schimperi* Sch.Bip. ex engl.	Harrittaa		HG	F	wh	Bleeding	Leaves are inserted into the nostrils to stop bleeding	N	
	*Xanthium strumarium* L	Bandaa	H	W	F	L/F r	Fungal on skin	The fruit or leaves of *X. strumarium* is rubbed and paint skin	D	TN010
	*Vernonia amygdalina* Del.	Ebichaa	T	W	F	L	Malaria	Crushed leaves of *V. amygdalina* concocted with leaves of *R. chalepensis*. One cup is served as a drink for 3–5 days with cold water in the morning	O	TN025
							Skin infection	Leaf of *V. amygdalina* is pounded and is washed by the plant or the leaf of is used as a soap to wash the infected body	D	
							Internal worm	The decocted leaves with 1cup of coffee for elders and half for children, 3–7 leave	O	
							Headache	Crushed leaves are put on head for 3 days 10 leaves	D	

Balanitaceae	*Balanites aegyptiaca* (L.) Del.c	Baddanna a	Sh	W	D	Gu m	Fibrile illness	Dried of the gum smoking and inhaled to nostril	N	TN012

Boraginaceae	*Cynoglossum lanceolatum* Forssk.	Maxxa annee	H	W	F	L	Febrile illness	Fresh leaf together with *O. lamiifolium i*s pounded and drunk with coffee or rub the body with the leaves	O	TN050
	*Cordia africana* Lam.	Wadde essa	T	HG	F	L	Toothache	Fresh leaf is chopped, chewed with salt and the juice is swallowed	O	TN069
					F	Fr	Headache	Whole plant is boiled in water then drunk and the head of the patient is washed with it or smell the plant as it	O	
	*Heliotropiu m cinerascens* steud. ex. DC.	Baalac abbii	H	W	F	L	Fibrile illness	Fresh leaves of water extract in one cup of tea into body or nasal	D	TN09
					F	L	Stomachache	Fresh leaves of *H. cinerascens* are extracted water taken into body by one cup of tea or coffee	O	

Brassicaceae	*Lepidium sativum* L.	Feecoo	H	HG	D	S	Malaria	Seed of *L. sativum,* bulb of *A. sativum* and rhizome of *Z. officinale* are pounded together and given to human with honey	D& O	TN027
					D	S	Tonsillitis	The seed of *L. sativum* and bulb of *A. sativum* are pounded together and given to human with honey	O	
					D	Fl	Tapeworm	Flowers are crushed, soaked in water for a day and drunk with alcohols	O	
	*Brassica carinata* A. Braun	Ija raafuu	H	HG	F	S	Stomach problem	Crushed and eaten with *A. sativum* by adding salt	O	TN038
					D	S	Cancer	The seed of *B. carinata* together with seed of *R. communis* crushed, powdered and mixed with honey and creamed body	O	

Carricaceae	*Carica papaya* L.	Pappay Aa	T	HG	D	S	Jaundice	Seed is roasted, pounded and is drunk two coffee cups every morning for 3 days	O	TN052

Casuarinaceae	*Casuarina cunninghamiana* Miq.	Shawes hawee	T	W	D/F	R	Lymphatic swelling	Concoction of fresh/dry root bark mixed with leaf of *C. macrostachyus* and water is given as drink	O	TN063
					F	R	Urine retention	The fresh root of *C. cunninghamiana* are mixed in water and the solution is taken	O	

Cucurbitaceae	*Cucumis ficifolius* A. Rich.	Accool iitoo	H	W	F	R	Wound	Root was crushed with in *S. araliacea* and put on wound surface	D	TN01
					F	R	Snake bite	Chewing the root and swallowing the juice	O	
	*Lagenaria siceraria* (molina) standl.	Buqqee	C	HG	F	S	Evil eye	Seeds are grounded and add to fire and smoke or drink with honey	Sm/O	TN015
					F	L	Snake bite	Fresh leaves are pounded and drunk in the presence of small amount of water	O	
	*Cucurbita pepo* L.	Debaaq ulaa	H	HG	F	L	Gastritis	The leaf cooked with *B. oleracea* then eaten 3–5 leaves	O	TN021
					D	S	Hookworm	Seeds are soaked in water over night, chew and swallowed as they are	O	

Euphorbiaceae	*Croton macrostachyus* Del.	Bakkan iisaa	T	W	F	L	Ascaris	The tip of fresh young leaf and the bark is pounded, boiled, add butter, cool it and after it solidifies, three nine tablets are made and three tablets for children, five to nine tablets for elders is given. Milk is drunk as an antidote	O	TN013
					F	L a	Ring worm	The latex of *C. macrostachyus* rubbed against the affected body part	D	
					F	L	Jaundice	Fresh leaves are cooked, pasted with honey and eaten as food	O	
					F	L	Gonorrhea	Seven, nine or eleven shoot tips is cut, cooked together with *R. chalepensis* and one spoonful of the solution is drunk per a day for seven consecutive days	O	
					F	B	Stomachac he	Fresh bark together with bulb of *A. sativum*, is pounded, mixed with butter and eaten	O	TN04
	*Euphorbia abyssinica* J.F.Gmel.	Adaam ii	T	W	F/D	L	Gonorrhea	Very small amount of the milky latex is mixed with red tef flour, bake and eaten for three consecutive days	O	
	*Ricinus communis* L	Qobbo o	H	HG	D	S	Impotency	Seeds are pounded, mixed with small quantity of latex from *Aloe spp.* And drunk one coffee cup before bed time for three days	O	TN055
	*Euphorbia tirucalli* L.	Qulqua lii	T	W	F	L a	Venereal disease	Latex is mixed with *E. tef* powder to prepare bread and given to eat	O	TN060

Fabaceae	*Vachellia etbaica* (schweinf.) Kyal. & Boatwr.	Doddot ii	T	W	F	L	Tonsillitis, gonorrhea	The fresh leaves of the *V. etbaica* is chewed and crushed, boiled and drunk	O	TN023
	*V. nilotica* (L.) P.J.H. Hurter & Mabb.	Qordi moo	T	W	D	St	Fibrile illness	The stem is dried and hot on the fire inhaled by nostril and smoked	Sm	TN057
	*V. oerfota* (forssk.) Kyal. & Boatwr	Ajoo	T	W	F	St	Toothache	Fresh stem of *V. oerfota* is chewed and holding on surface of teeth	O	TN06
	*Calpurnia aurea* baker	Ceekat aa	Sh	W	F	st	Syphilis	The seeds are crushed, mixed with honey and half teaspoon is eaten for three consecutive days	O	TN016
	*V. abyssinica* (Hochst. ex Benth.) Kyal. & Boatwr.	Laafto	T	W	F	L	Back-pain	Fresh crushed leaves are mixed with water and drunk	O	TN045
	*Trigonella foenum- graecum* L.	Abishii	H	HG	D	S	Gastric	The powder of *T. foenum-graecum* is mix with water, and drink by glass	O	TN03
					D	S	Body swelling	The seed of *T. foenum-graecum* is crushed, powdered, mixed with honey and little water then boiled like “porridge” and eaten	O	

Salicaceae	*Dovyalis abyssinica* (A.Rich.Wa rb)	Kooshi moo	Sh	W	F	Fr	Intestinal parasites	Fruit is eaten as food for the case of intestinal parasite before breakfast every morning	O	TN043

Lamiaceae	*Ocimum lamiifolium* Hochst. ex Benth.	Damm akesse	Sh	W	F	L	Febrile illness	Fresh leaf of *O. lamiifolium* together with leaf of *E. globulus*, is pounded, mixed with water and drunk or inhale the vapor of the boiled mixture	O	TN018
					F	L	Headache	Leaf of *O. lamiifolium* is massaged and sniffed	O	
	*Clerodendrum myricoides* R. Fern	Marasii saa	Sh	W	D	R	Evil eye	Root is inserted on fire and the smoke is inhaled through the nostrils	N	TN049
					F	R	Snake bite	Fresh root decoction is drunk immediately after the bite	O	
					D	R	Malaria	Root together with the root of *W. somnifera* are pounded, mixed with water, boil, cool and then drunk	O	
	*Ajuga integrifolia* Buch.	Harma gussaa	H	W	D/F	L	Body swelling	Dry or fresh leaves are pounded & drunk or tied on the swelling	D	TN032

Linaceae	*Linum usitatissimum* L.	Talbaa	H	HG	F	S	Wound	Seed of *L. usitatissimum* is pounded mixed with honey and creamed on wounded part	D	TN067
					D	S	Amoeba	The pounded seed is mixed with water and drunk before breakfast	O	

Meliaceae	*Melia azedarach* L	Niimii	T	HG	F	St	Toothache	Young stem is chewed and kept on the teeth	O	TN051

Francoaceae	*Bersama abyssinica* Fresen.	Azamir	Sh	HG	D	L/R	Hypertensi on	The dry of leaf and root are powdered, mixed with water and drunk	O	TN08
					D	L/R	Cough & ascaris	The leaf and root are crushed, powdered, boiled with milk and drunk	O	
		Lolchii saa	Sh	W	D/F	R	Syphilis	Fresh root is chewed and swallow its content, root is crushed, boil and drunk for three days	O	TN046
					F	B	Ascaris	Fresh bark is pounded, pasted with honey and is eaten	O	

Moringaceae	*Moringa stenopetala* (Bak.f.) Cufod.	Shifera	T	W	F	L	Hypertension	The part of leaf is squeezed then drink/crush then boil, filtrate, and drink	O	TN064

Myrtaceae	*Eucalyptus globulus* Labill.	Barzaaf ii adii	T	W	F	L	Cough	Fresh young leaves are boiled in water and fumigate the vapor under sealed clothes at bed time	Sm	TN011

Olacaceae	*Ximenia americana* L.	Hudha a	Sh	W	F	L	Toothache	Bark of the root is chewed and kept it for a while between the teeth	D	TN037
					D	L/R	Nasal bleeding	The fine powder of the root is mixed with the powder of the leaf of *A. africanus* and is applied	O	

Oleaceae	*Olea europaea subsp.cuspi data* (Wall. Ex G.Don) Cif.	Ejersa	T	W	F	L	Itchy skin	Leaf together with dried leaf of *Chenopodium myricoides* is boiled in water and steam the vapor to the part	O	TN026

Phytolaccaceae	*Phytolacca dodecandra* L.'Herit.	Hando odee	S	W	F	R	Rabies	Root together with root of *A. abyssinica*, *J. schimperiana* is pounded, mixed with water; one katicala glass of the solution is given at interval of seven day	O	TN031

Poaceae	*Cymbopogo n citratus* (DC.) Stepf	Tajiisar	H	HG	D	R	Evil eye	The roots of *C. citratus* with root of *C. macrostachyus* are pounded dried put on fire and the smoke is inhaled	Sm	TN066
					D	L	Dandruff	The leaf is crushed powdered mixed with butter and creamed on affected part	D	

Ranunculaceae	*Nigella sativa* L.	Abasuu da	H	HG	D	S	Depression	Dried the powder of seed is added to tea and drunk to stimulate mental	O	TN02
		Gurraac ha			D	S	Tonsillitis	The seed of *N. sativa* is pounded powdered and added to coffee. Then drunk for 3-4 consecutive days	O	
	*Clematis simensis* Fresen.	Hida fiitii	H	W	F	L	Hemorrhoi ds	Pounded/crushed leaf will be tied on the affected area of the skin	D	TN034

Rhamnaceae	*Ziziphus spina-christi* (L.) Desf.	Qurqur aa	T	W	D	L/R	Evil eye	Dried leaf or root is used for fire fumigation and inhalation	D	TN061
	*Rhamnus prinoides* L.Herit.	Geesho o	Sh	HG	F	L	Tonsillitis	Chew the leaf and swallow twice a day for three days	D	TN029

Rubiaceae	*Coffea arabica* L.	Buna	Sh	HG	D	S	Diarrhea	Powder of roasted coffee bean is mixed with butter and eaten or drunk before breakfast for 3/4 days	O	TN014
					D	S	Fire burn	Seed of *C. arabica* is roasted, pounded, powdered and applied on affected part	N	
					D	S	Spider poison	Seed of *C. arabica* is roasted, powdered, mixed with butter and painted	O	
					D	S	Eye disease	Roasted seeds of *C. arabica* is pounded together with Leaf of *T. foenum graecum*, mixed with butter and rubbed on the external eye	O	

Rutaceae	*Citrus x limon* (L.) Osbeck	Loomii	Sh	HG	F	Fr	Athletes foot	The fruit of *C. limon* is squeezed and creamed on affected for continuous days	D	TN047
					D	L	Cough	The leaf of *C. limon* is pounded, powdered, mixed with milk and boiled and added sugar then drink pure liquid during feeling pain	O	
	*Ruta chalepensis* L.	Xeenaa daamii	Sh	HG	F	L	Fever	Fresh leaf is pounded together with *Z. officinale*, added to coffee and then a cup of coffee is drunk every morning for three consecutive days	O	TN070
					F	L	Headache	Fresh leaf together with *Z. officinale* is pounded, add to coffee and drunk	O	
					F	L	Evil eye	Fresh leaf together with leaf of *D. stramonium* is rubbed on the body of the patient or washes with the solution of these plants	D	

Sapindaceae	*Dodonaea viscosa* L.f.	Itacha	Sh	W	D	L	Ear wound	Crushed, mixed with butter and placed on the damaged part	D	TN040

Scrophulariaceae	*Verbascum sinaiticum* Benth.	Gurraa harree	H	W	F	L	Diarrhea	The fresh leave is crushed, homogenized in water and drunk by half of cup tea	O	TN030
					F	L	Hemorrhag e	The leave of *V. sinaiticum* is crushed, homogenized in water and drunk	O	

Simaroubaceae	*Brucea antidysenterica* Fresen.	Qoman yoo	Sh	W	D	L	Wound	Dried leaf of *B. antidysenterica* is pounded, mixed with butter and creamed body	D	TN056

Solanaceae	*Datura stramonium* L.	Manjii	H	W	F/D	L	Cough	Dried or fresh leaf together *W. sominifera* and *L. tomentosa*, are pounded, half spoon is added to a cup of coffee and drunk every morning until recovery	O	TN048
					F/D	S	Toothache	Seeds are boiled in water and inhaled the vapor	D	
					F	L	Eye disease	Leaf is squeezed and the juice is applied to the eye	Ey	
	*Solanum incanum* L.	Hiddii	Sh	W	D	R	Snake bite	Root powder is drunk with coffee	O	TN036
					F	R	Toothache	Root is chewed and keeps between the teeth	O	
	*Solanum marginatum* L.f.	Hiddii	Sh	W	F	Fr	Bleeding	Fruit and/or leaf is pounded and tie on the bleeding part	D	TN035
					F	St	Wound	Warm the stem on fire and placed it on the head again and again	D	
					F	L	Nasal bleeding	The leaf is chopped and placed in the nostrils or small amount of the fruit juice is applied through the nostrils	N	
					F	Fr	Leech	Fruit is squeezed and the juice is mixed with milk and applies through the nostrils	D	
	*Capsicum annuum* L.	Qaaraa	H	HG	D	Fr/S	Skin rash	Fruit and seed of *C. annuum* is pounded, powdered mixed with butter and creamed the infected part	D	TN053
					D	Fr/S	Tonsillitis	Fruit and seed of *C. annuum i*s pounded, powdered, mixed with oil, roasted and drunk	O	
	*Withania somnifera* (L.) Dunal in DC.	Daadh Oo	Sh	W	F	L	Evil eye	The leaf is squeezed with leaves of *R. chalepensis* and drunk 1 soup spoon	O	TN017
					F	L	Insect allergy	Crushed and tied by cloth in the area	O	
	*Lycopersicon esculentum* Mill.	Tiimaa tiimii	H	HG	F	S	Eye disease	The seed of *L. esculentum* is eaten	O	TN072

Urticaceae	*Urtica simensis* Hochst. ex. A.Rich.	Doobii	H	W	F	L	Gastritis heart disease	Eat in the form of stew (“wot”) against gastritis & heart disease	O	TN073
					D	R/L	Gonorrhea	The root and leaves of *U. simensis* with the bark of *C. macrostachyhus* are pounded, powdered, mixed with little water, filtered, then a cup of filtrate is drunk for 5 days in the morning	O	

Verbanaceae	*Lippia adoensis* var. adoensis Hochst. ex Walp.	Kusaay ee	S	W	D	R	Intestinal parasite	Fresh or dried root together with the dried bark of *C. macrostachyus* is crushed and eaten after breakfast	O	TN044
					D	L	Cough	Dried leaf is pounded and boiled and a tea spoon of it is added to cup of coffee and drunk for three days every	O	

Vitaceae	*Cyphostemma cyphopetalum* (Fresen.) Desc. ex wild & R.B.Drumm.	Gaalee waraab essa	Cl	W	F	Fl	Hemmoroi ds	Fresh flower is squeezed and the juice is applied or the flower is rubbed over the wound	D	TN028

Zingiberaceae	*Zingiber officinale* R oscoe	Ziinjibi laa	H	HG	D	R	Stomachac he	Chew and swallow the juice/drink with tea	O	TN071
					F	L	Body swelling	The fresh of leave is chew and tie on surface part of swelled body	O	

Hb = habit; Pu-part used; Ut = used to treat; Cp = condition of preparation; RA = route of administration; T = tree; H = herb; Sh = shrub; Cl = climber; Hu = human; F = fresh; D = dry; F/D = fresh and dry; O = oral; De = dermal; Na = nasal; Ey = eye; Er = ear; L = leaf; R = root; Sm = smoke; St = stem; Ba = bark; Fl = flower; Fr = fruit; S = seed; Bu = bulb; La = latex; Wp = whole plant; Ht = habitat; HG = home garden; W = wild; VN = voucher number.

**Table 3:** Frequently cited medicinal plants of Zuway Dugda district.

Scientific names	Frequency of citation	Percentage
*Allium sativum* L.	52	52
*A. pubescens*	54	54
*Balanites aegyptiaca* (L.) Del.	41	41
*Carissa spinarum* L.	45	45
*Cordia africana* Lam.	39	39
*Croton macrostachyus* Hochst. ex Del.	43	43
*Cucumis ficifolius* A.Rich.	44	44
*O. lamiifolium*	57	57
*Ruta chalepensis* L.	50	50
*Withania somnifera* (L.) Dunal	53	53

**Table 4:** Informant consensus factor by categories of diseases in the study area.

No	Categories	No of species	No of use citation	ICF
1	Evil eye and evil sprit	10	29	0.68
2	Headache, toothache, depression, febrile illness, and fever	19	28	0.33
3	Skin infection, ring worm, skin rash, dandruff, and athletes foot	5	42	0.90
4	Stomachache, Ascaris, intestinal parasites, amoeba, diarrhea, gastric, and tape worms	17	27	0.38
5	Body swelling and lymphatic swelling	5	16	0.73
6	Tonsillitis, problem of throat	6	18	0.71
7	Malaria, spider poison, and insect allergy	8	23	0.68
8	Rabies, jaundice, cold, cough, pneumonia, and rheumatism	10	19	0.5
9	Snake bite	4	46	0.93
10	Wound, fire burn, ear wound, bleeding, and nasal bleeding	16	34	0.55

**Table 5:** Preference ranking of medicinal plants used to treat wound in Zuway Dugda district.

Medicinal plants	Respondents (R_1–10_)										Total	Rank
	R1	R2	R3	R4	R5	R6	R7	R8	R9	R10		
*Achyranthes aspera* L.	3	2	3	5	4	4	3	2	4	4	34	4^th^
*A. africanus*	4	5	4	5	3	4	3	5	5	4	42	1^st^
*Brucea antidysenterica* J.F.	3	4	3	5	3	3	2	2	4	3	32	6^th^
*C. spinarum*	4	3	4	5	5	4	2	4	3	5	39	3^rd^
*C. ficifolius*	3	2	4	2	3	3	4	3	5	4	33	5^th^
*Dodonaea viscosa* Jacq.	4	3	4	3	5	3	5	4	5	4	40	2^nd^
*Linum usitatissimum* L.	4	2	4	3	2	3	1	4	4	3	30	7^th^
*S. marginatum*	4	4	1	3	2	2	3	3	1	4	25	8^th^

**Table 6:** Paired comparison of medicinal plants used to treat febrile illness (R stands for informants).

Medicinal plants	Respondents R (1–7)							Total	Rank
	R1	R2	R3	R4	R5	R6	R7		
*Vachellia nilotica* (L.) P.J.H	3	2	2	3	4	3	2	19	4^th^
*B. aegyptiaca*	3	4	4	3	5	4	3	26	2^nd^
*C. lanceolatum*	3	2	3	2	3	2	2	17	5^th^
*Heliotropium cinerascens* Aitch.	3	2	4	2	3	2	4	20	3^rd^
*O. lamiifolium*	4	5	4	5	3	4	5	30	1^st^

**Table 7:** Direct matrix ranking for multipurpose uses of medicinal plants.

Use categories	Name of species									
	*V. oerfota*	*E. globulus*	*C. papaya*	*C. macrostachyus*	*C. spinarum*	*V. etbaica*	*V. nilotica.*	*V. abyssinica*	*O.europaea subsp.cuspidata*	*C. africana*
Medicine	4	4	4	5	5	3	4	4	5	5
Edible fruits	0	0	5	0	0	0	0	0	0	3
Charcoal	4	4	0	4	2	5	4	5	5	4
Construction	2	4	0	4	3	4	5	5	5	5
Fences	4	3	1	4	4	5	4	5	4	4
Fire wood	4	4	2	4	3	4	5	4	5	5
Total	18	19	12	21	17	22	22	23	24	26
Rank	8^th^	7^th^	10^th^	6^th^	9^th^	4^th^	4^th^	3^rd^	2^nd^	1^st^

**Table 8:** Ranking of medicinal plants reported as threatened in the study area.

Major threats	Respondents R (_1–10_)										Total	%	Rank
	R1	R2	R3	R4	R5	R6	R7	R8	R9	R10			
Agricultural expansion	4	5	4	4	4	5	5	4	4	5	44	19.13	1^st^
Fire wood	4	5	3	4	4	3	2	3	4	5	37	16.1	4^th^
Charcoal production	4	4	5	3	4	4	4	5	3	5	43	18.7	2^nd^
Construction	4	4	2	3	4	4	5	4	3	3	39	17.0	3^rd^
Over grazing	3	5	4	3	2	2	4	3	4	4	34	14.8	5^th^
Drought	4	4	3	4	4	2	4	3	3	2	33	14.35	6^th^

## Data Availability

The data used in this study are available from the corresponding author upon request.
